# iMAR: An Interactive Web-Based Application for Mapping Herbicide Resistant Weeds

**DOI:** 10.1371/journal.pone.0135328

**Published:** 2015-08-12

**Authors:** Silvia Panozzo, Michele Colauzzi, Laura Scarabel, Alberto Collavo, Valentina Rosan, Maurizio Sattin

**Affiliations:** 1 National Research Council (CNR)—Institute of Agro-environmental and Forest Biology (IBAF), Legnaro (PD), Italy; 2 Free-lance webmaster, Padova, Italy; University of Illinois at Urbana-Champaign, UNITED STATES

## Abstract

Herbicides are the major weed control tool in most cropping systems worldwide. However, the high reliance on herbicides has led to environmental issues as well as to the evolution of herbicide-resistant biotypes. Resistance is a major concern in modern agriculture and early detection of resistant biotypes is therefore crucial for its management and prevention. In this context, a timely update of resistance biotypes distribution is fundamental to devise and implement efficient resistance management strategies. Here we present an innovative web-based application called iMAR (interactive MApping of Resistance) for the mapping of herbicide resistant biotypes. It is based on open source software tools and translates into maps the data reported in the GIRE (Italian herbicide resistance working group) database of herbicide resistance at national level. iMAR allows an automatic, easy and cost-effective updating of the maps a nd provides two different systems, “static” and “dynamic”. In the first one, the user choices are guided by a hierarchical tree menu, whereas the latter is more flexible and includes a multiple choice criteria (type of resistance, weed species, region, cropping systems) that permits customized maps to be created. The generated information can be useful to various stakeholders who are involved in weed resistance management: farmers, advisors, national and local decision makers as well as the agrochemical industry. iMAR is freely available, and the system has the potential to handle large datasets and to be used for other purposes with geographical implications, such as the mapping of invasive plants or pests.

## Introduction

The new European legislation on the sustainable use of pesticides aims to significantly decrease the impact of crop protection practices on the environment and human health by reducing the use and risks of plant protection products [1]. To reach this goal, more science-based information should be conveyed to farmers and other stakeholders through a better use of Information and Communication Technology (ICT) tools targeting complex socio-ecological systems [2, 3].

A number of studies have highlighted the economic and environmental cost of weeds [4]. More specifically, the fast evolution and worldwide spread of pesticide resistance can jeopardize the sustainability of many cropping systems, as well as vegetation management in non-agricultural areas, and have a significant adverse impact on the environment [5–8]. Among pesticides, herbicides are the most difficult to substitute with alternative methods [9] due to the ubiquitous nature of weed infestations, so chemical control is still the most common and effective way to prevent yield losses in crops and to control weeds in non-agricultural areas [10, 11]. Globally, herbicides account for up to 50% of the plant protection market [12]. Where resistance is not present, herbicides are relatively cheap tools in relation to other available options, avoid labor-intensive and time-consuming soil cultivation practices, and ultimately result in cost-effective, safe and profitable food production [13]. On the other hand, the high phenological and genetic variability among weeds, together with an over-reliance on herbicide use, frequently results in the selection of herbicide-resistant weed populations, and 86 crops in 66 countries are now affected worldwide [14]. Unfortunately, resistance to herbicides that have a low environmental impact is particularly common [15, 16] due to their specific metabolic target [17] and has become one of the major concerns for weed management and more generally for sustainable crop production. To date, 236 weed species (138 dicots and 98 monocots) worldwide have evolved resistance to different herbicide Sites of Action (SoA) [14].

A resistant biotype is considered to be a group of individuals that share several physiological characteristics, including the ability to survive one or more herbicides belonging to a particular group (see HRAC herbicide classification [18]) used at a dose that would normally control them. Each biotype is therefore characterized by a certain resistance pattern, i.e. number and type of herbicide SoA to which it is resistant. The mapping of these resistant biotypes, continuously updated and promptly disseminated, is important to efficiently manage and possibly prevent the evolution of herbicide resistance. This information can be useful to various stakeholders: farmers, advisors, national and local decision makers as well as the agrochemical industry. Large-scale maps showing the spreading of resistance at global and continental level according to the SoA are already available in the world database of herbicide resistance [14]; however mapping systems capable of generating and easily updating more detailed maps are needed at national, regional and local level.

A lot of work has been done on weed mapping, mainly related to a) the distribution of different weed species (e.g. WeedMapper provides a collection of confirmed noxious weed sites across Oregon State, see http://www.compasstoolsinc.com/weedmapping.htm, or in North Dakota the North Dakota Department of Agriculture provides a similar service on the Government website, see https://apps.nd.gov/ndda/mapping/) and especially invasive ones [19], and b) spatial distribution of weeds at single field level [20–22]. In Europe, a working group of the European Weed Research Society (EWRS, Weed Mapping Working Group, http://www.ewrs.org/weed_mapping.asp) is actively pursuing its mission to provide an overview on the occurrence and spreading of weeds in Europe [23]. Concerning herbicide resistance mapping, just a few examples are reported in the literature and they deal with limited areas or with specific weed species. Bayer Crop Science has reported the geographic distribution of resistance to a specific group of herbicides (i.e. acetyl-CoA carboxylase—ACCase—inhibitors) concerning more than 2500 weed biotypes of some of the most troublesome weeds in Europe, such as blackgrass (*Alopecurus myosuroides*), ryegrass (*Lolium multiflorum*) and silky-bent grass (*Apera spica-venti*), collected in France, Germany and Great Britain [24]. Similar data were used to study the geographic distribution of resistance-endowing mutations within the gene encoding the plastidic ACCase in *A*. *myosuroides* in France [25]. Very recently, Massa et al. [12] developed a geo-referenced database (Weedscout 2.0) in which the distribution of herbicide-resistant *A*. *spica-venti* populations is mapped at European level, but it is currently not available online. To our knowledge all these examples refer to a specific time and are not promptly updated and made publicly available.

**Fig 1:**
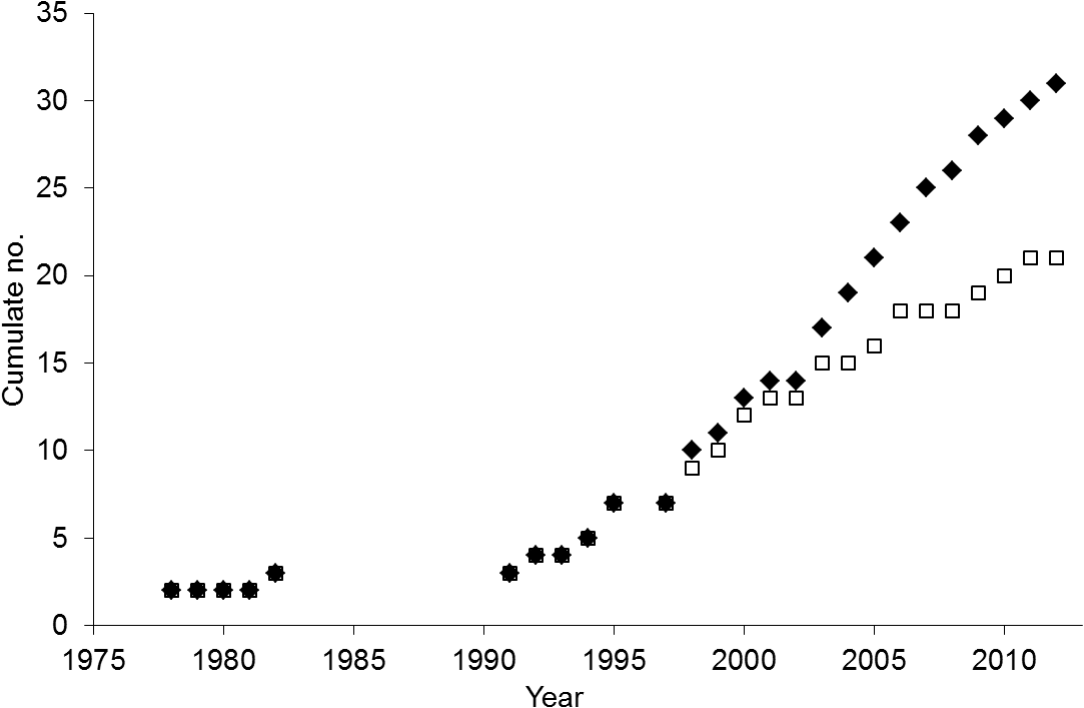
The chronological increase in the number of herbicide-resistant species (□) and biotypes (♦) in Italy.

In 1997, when the number of resistance cases in Italy began to increase significantly [26, 27], the Italian herbicide resistance working group (GIRE) was founded. The main goals of GIRE are to improve cooperation and communication between public and private researchers and involved stakeholders in order to monitor the evolution of herbicide resistance all over the country and to provide and efficiently communicate resistance status and management strategies. To promote the dissemination of information, GIRE created a website in 2009 [28] that includes the updated situation of herbicide resistance in Italy, the main biological traits of the weeds involved, general and specific resistance management guidelines, literature and news. The website currently has about 4,000 contacts per month. Given that the number of weed species and resistant biotypes involved has been steadily increasing (Fig 1) and now affect the most important arable as well as perennial cropping systems [26, 29–33], it has become a priority to promptly communicate the status of herbicide resistance throughout the country.

## Background

The need for mapping herbicide resistance was recognized early. The first Italian herbicide resistant weed populations appeared about 35 years ago when cases of resistance to atrazine were identified in three weeds (*Solanum nigrum*, *Amaranthus* spp. and *Chenopodium album*) infesting maize crops [34]. The distribution of these populations was reported on a hand-drawn map [35]. Then, in 1998 the spreading of resistant biotypes was reported on geographic maps extracted from the encyclopedia Encarta [26]. Thereafter, the number of resistant cases, weeds and cropping systems involved increased rapidly (Fig 1) resulting in the need for a better system to design resistance maps. Therefore, from 2004 onwards, maps were generated using the free software ArcExplorer 2 and the Italian map of municipalities provided by the Italian National Institute of Statistics (ISTAT). The distribution of each resistant biotype was obtained by changing the color of the territory of the municipalities where at least one resistant population had been confirmed.

To date, 31 resistant biotypes have been documented in Italy (including 5 multiple resistant) involving 21 species (6 dicots and 15 monocots). Most cropping systems (wheat, maize, rice, soybean and perennial crops) are involved and it is estimated that more than 200,000 ha are infested by resistant populations [28]. Such complexity soon revealed the limits of the mapping system based on ArcExplorer 2. The data input, done using Excel sheets, was highly error-prone and resistance maps were tricky to obtain, had to be manually uploaded into the website and were difficult to update. Finally, the system appeared obsolete when the need to consider multiple resistance (i.e. weed populations resistant to herbicides with different SoA) became compelling.

A relevant issue for large-scale mapping tools is related to the costs of base maps and software applications that often limit the development and free access to such tools [36]. Nevertheless, the tremendous work, both theoretical and programming, undertaken by the developers of open source tools in the last decade has made GIS and map tools available for the development of powerful and cheap web systems [37].

This paper presents an interactive web-based application for mapping herbicide resistant weeds that is based on open source software (OSS) and maps. The application, named iMAR (interactive MApping of Resistance), makes use of the GIRE database of herbicide resistant populations in Italy and is publicly available on the GIRE website.

## The Interactive MApping of Resistance (iMAR) Web-Based Application

**Fig 2:**
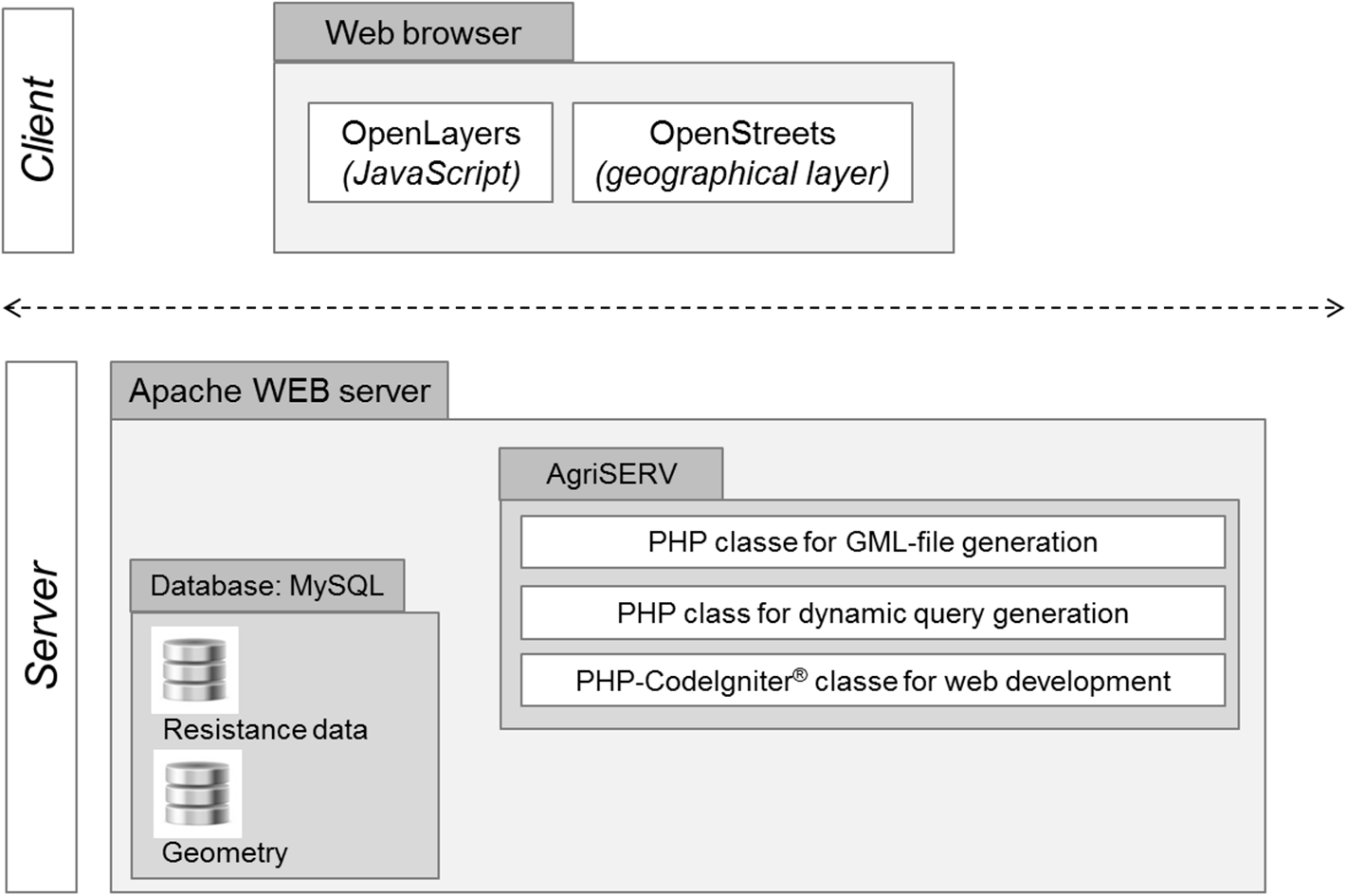
Architecture of the iMAR application.

The innovative iMAR web application (http://5.135.187.175/agri_test/index.php/mappe/pagedef/IT) allows customized maps to be created of herbicide resistance distribution at national level. It is part of the GIRE website [28] and is installed on a “LAMP” server. LAMP is the acronym for a popular open source web platform commonly used to run dynamic websites and servers. It includes Linux, Apache, MySQL and PHP and is considered the best platform for the development and deployment of high performance web applications that require a solid and reliable foundation (Fig 2). Other specific software (mainly PHP and JavaScripts) have also been developed for user interaction, dynamic query and vectorial data preparation (GML format) for maps realization. The process of map visualization takes place in the GIS client, which runs within a web browser (Fig 2) and is based on a JavaScript library called OpenLayers. OpenLayers (http://openlayers.org/) implements a constantly developing JavaScript Application Programming Interface (API) for building rich web-based geographic applications with no server-side dependencies. Although it behaves similarly to API's such as Google Maps and MS Bing Maps, OpenLayers is open source, which means it is free for modification and redistribution. Resistance maps are drawn by OpenLayers on a geographic layer provided by OpenStreetMap (https://blog.openstreetmap.org), which is an open initiative to create and provide free geographic data such as street maps. In computer terms OpenStreetsMap is an XML database where the raw data describing the positions of roads, rivers, towns, points of interest, etc. are stored. In our context it provides the base layer for the maps and is used as an alternative to Google Maps, releasing GIRE from onerous user costs [36]. Currently, the database on which iMAR is based contains only Italian data, but the system could include larger datasets, for example data at European scale.

The system OpenLayers + OpenStreetMap works on the client side and provides basic functions such as navigating the map and switching visible layers. The fact that the system has been developed entirely with the use of open source technology, makes it both cost-efficient as well as fully adaptable to most hardware and software configurations. It has been optimized for Firefox and Chrome browsers and to run on a Microsoft Windows operating system (Windows 7 or later). iMAR is the property of a public research institution (CNR—National Research Council of Italy) that also manages/inputs the database of resistant populations, i.e. CNR has control of all the steps, it is freely available, is not related to any commercial interest and is based on an original approach.

### Data input

Data inputs for the iMAR are obtained from GIRE‘s collection of Italian herbicide resistant weed populations, which includes more than 1700 entries. All putative resistant populations collected throughout the country are tested for resistance at the CNR Institute of Agro-environmental and Forest Biology (IBAF) through standardized whole plant herbicide bioassays conducted in a glasshouse. The description of materials and methods used in the bioassays for the various weed species are detailed in several published papers [26, 29, 31, 32, 38–41]. It should be mentioned that a population is ascribed as resistant (R) to a herbicide when more than 20% of treated plants survived the recommended herbicide field dose [27].

**Fig 3:**
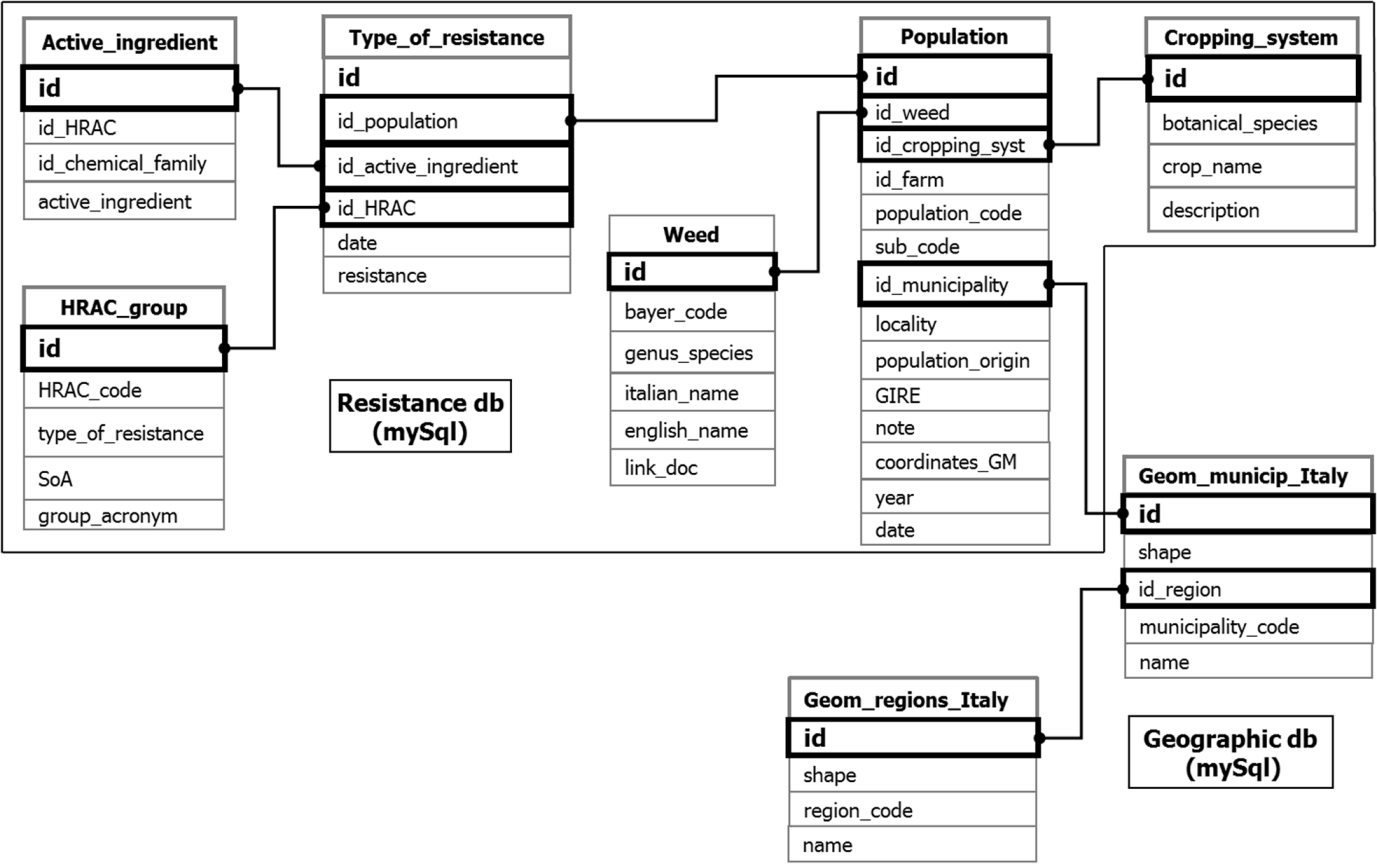
Most relevant tables and relations of the two mySQL databases (Resistance db and Geographic db) on which iMAR is based.

The mySQL database with populations data (resistance db) includes six tables (Fig 3). Tables “Type of resistance” and “Populations” contain data strings that include all information about the populations (identification code, weed species, cropping system involved, population origin and municipality where it was collected) and the results of resistance assessments for each herbicide tested. For some species (e.g. weed species with high rate of hybridization as *Lolium* and *Amaranthus*, or weed species difficult to identify as *Echinochloa*) only the genus followed by indication of multispecies (spp.) is reported.

In order to describe the resistance pattern of a population tested with N herbicides, its identification code (id) is repeated N times in the “Type_of_resistance” table. Tables “Active_ingredient” and “HRAC_group” contain the information for classifying the herbicides according to their SoA and to create maps per type of resistance, whereas tables “Weed” and “Cropping_system” work in the same way to design maps per weed species and cropping system, respectively. All this information identifies a population as unique, which is geo-localized through the geographic database (Fig 3) that contains the geometric information (physical boundaries) of Italian regions and municipalities based on a bitmap image provided by the Italian National Institute of Statistics (ISTAT). The geographic and resistance databases are related through the identification codes of the municipalities (id_municipality).

### Map generation

The map generation system involves sequential steps in which the software tools interact with each other under the coordination of a PHP software-code, specifically developed within the CodeIgniter framework.

According to the query launched by the user, a number of layers equal to the number of type(s) of resistance involved (i.e. R_1_, R_2_, …, R_k_) are created and overlapped and, per each R_k_ type of resistance, the M_1_, M_2_, …, M_n_ municipalities affected are identified via their id. A specific procedure then retrieves and transforms the *x*, *y* arrays of the border coordinates of each municipality into GML files. These are stored in the geographic database using the spatial extension which, besides efficient storage, enables the generation and analysis of geographic features.

The procedure generates *k* files (one file per resistance type). On the client side, each GML file is projected by OpenLayers using a Spherical Mercator projection (EPSG:3857), which is also used by Google Maps (http://docs.openlayers.org/library/spherical_mercator.html).

The distribution maps of each resistant biotype are obtained by changing the color of the territory of the municipalities where at least one confirmed resistant population had been collected. Therefore municipalities with different numbers of resistant populations will appear with the same color. This, together with the nature of the monitoring done by GIRE, which is based on a) end users complaints about herbicide failure and b) priority given to samples collected in municipalities where herbicide resistance had not previously been reported, makes the output maps “qualitative” because they do not provide reliable information on the spread of resistance within each municipality.

### Output

**Fig 4:**
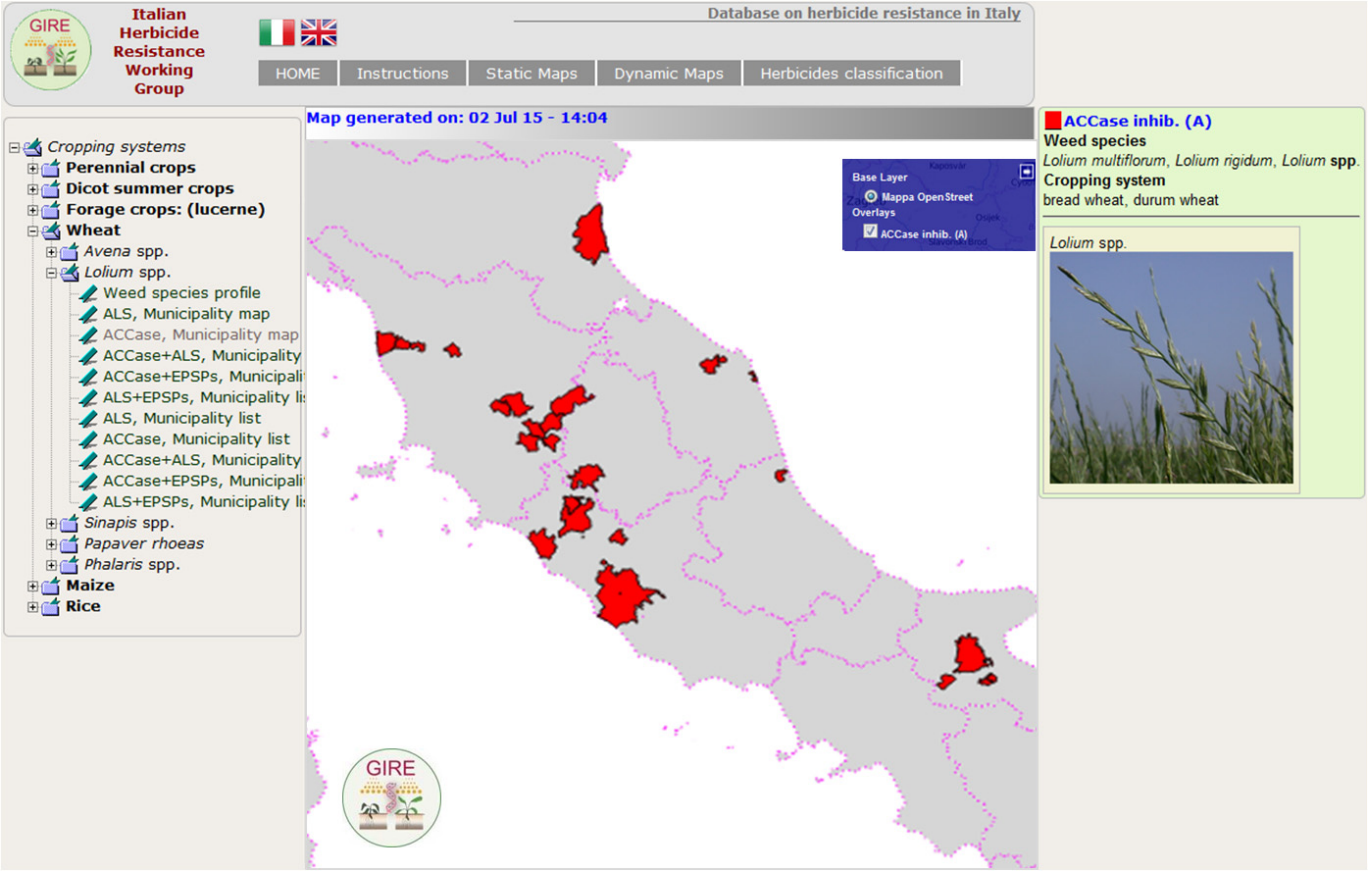
Example of resistance map built using the “static” system. On the left the tree menu for choosing the features (crop system and weed species) and on the right the results of the selection. Municipalities where at least one case of resistance to ACCase inhibitors is reported in red. Note: this image is representative of the OpenStreetMap-generated image; for a real representation of this map generated by iMAR go to the link: http://5.135.187.175/agri_test/application/views/istruzioni_Fig1.php

Two mapping systems with different levels of complexity, called “static” and “dynamic”, currently coexist in the iMAR application. The “static” system (http://5.135.187.175/agri_test/index.php/mappe_stat/gire/) is simple and intuitive and is intended for website visitors who are not computer/internet experts. Maps are created according to the cropping system affected by herbicide resistance in Italy. A hierarchical tree menu guides users through the choice of the cropping system and then of the weed species of interest (Fig 4). Along with the resistance map and the corresponding list of municipalities affected, the output includes the weed card, i.e. the page that describes the biological traits of the weed species.

**Fig 5:**
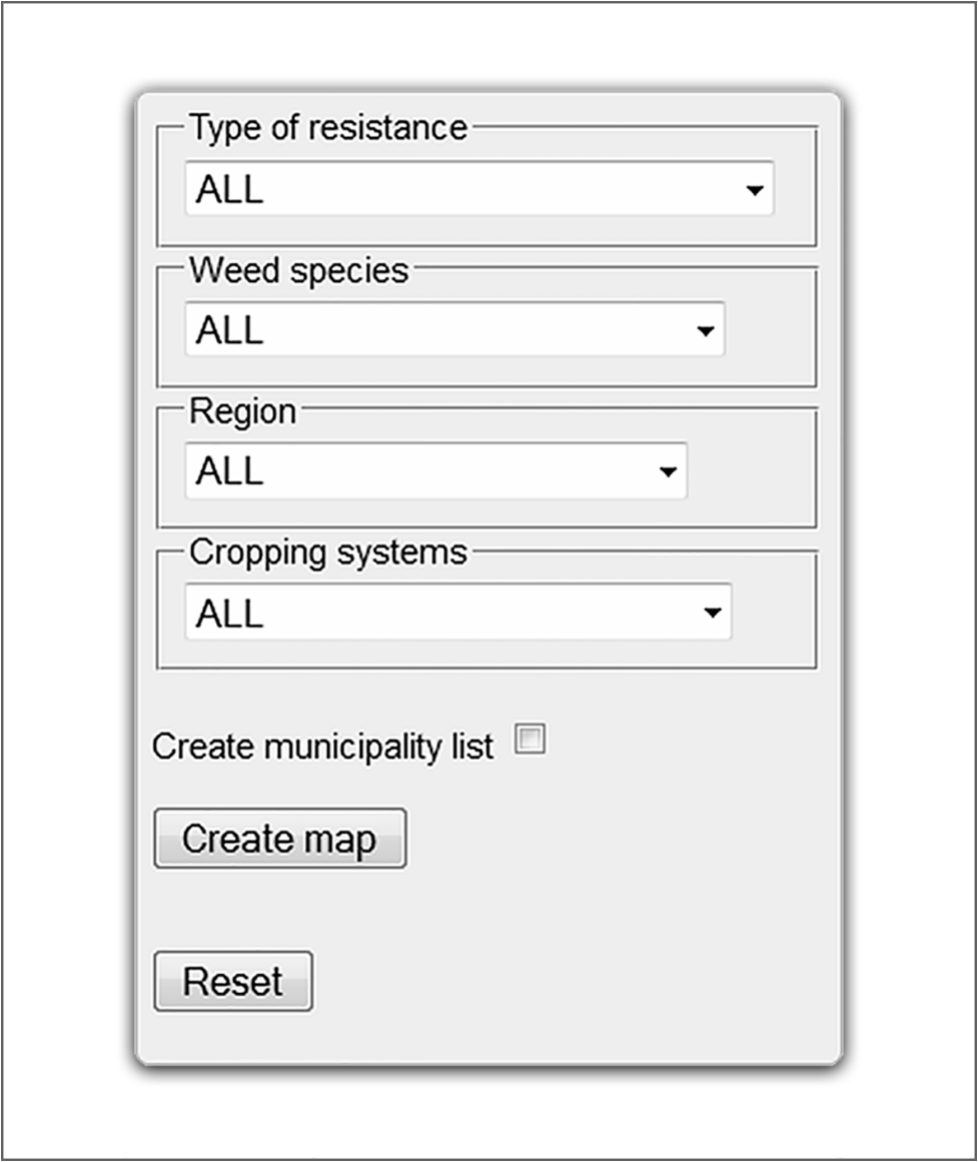
The drop-down menu of the “dynamic” system. It is possible to visualize general maps for each field leaving “ALL” and clicking the “Make map” button. Otherwise the search field can be limited to visualize only the samples of a “Weed species” resistant to a “Type of resistance” in an Italian “Region” in a specific “Cropping system”. It is also possible to visualize the list of municipalities involved selecting the appropriate box (i.e. “Generate municipality list”) before clicking the button “Make map” and, finally, to “Reset” all fields before starting a new interrogation.

To allow the user a greater flexibility and to better exploit the data contained in the resistance database, a more “dynamic” system has been devised based on four criteria (http://5.135.187.175/agri_test/index.php/mappe/gire/). Through a multiple query including four drop-down menus (Fig 5), the user is guided to generate the desired map by selecting the type of resistance (e.g. ACCase inhibitors, ALS inhibitors), weed species, Italian region and cropping system of interest. For each criterion the option “all” is also available and this allows complex maps to be created that include more types of resistance and/or more weed species and/or more cropping systems.

**Fig 6:**
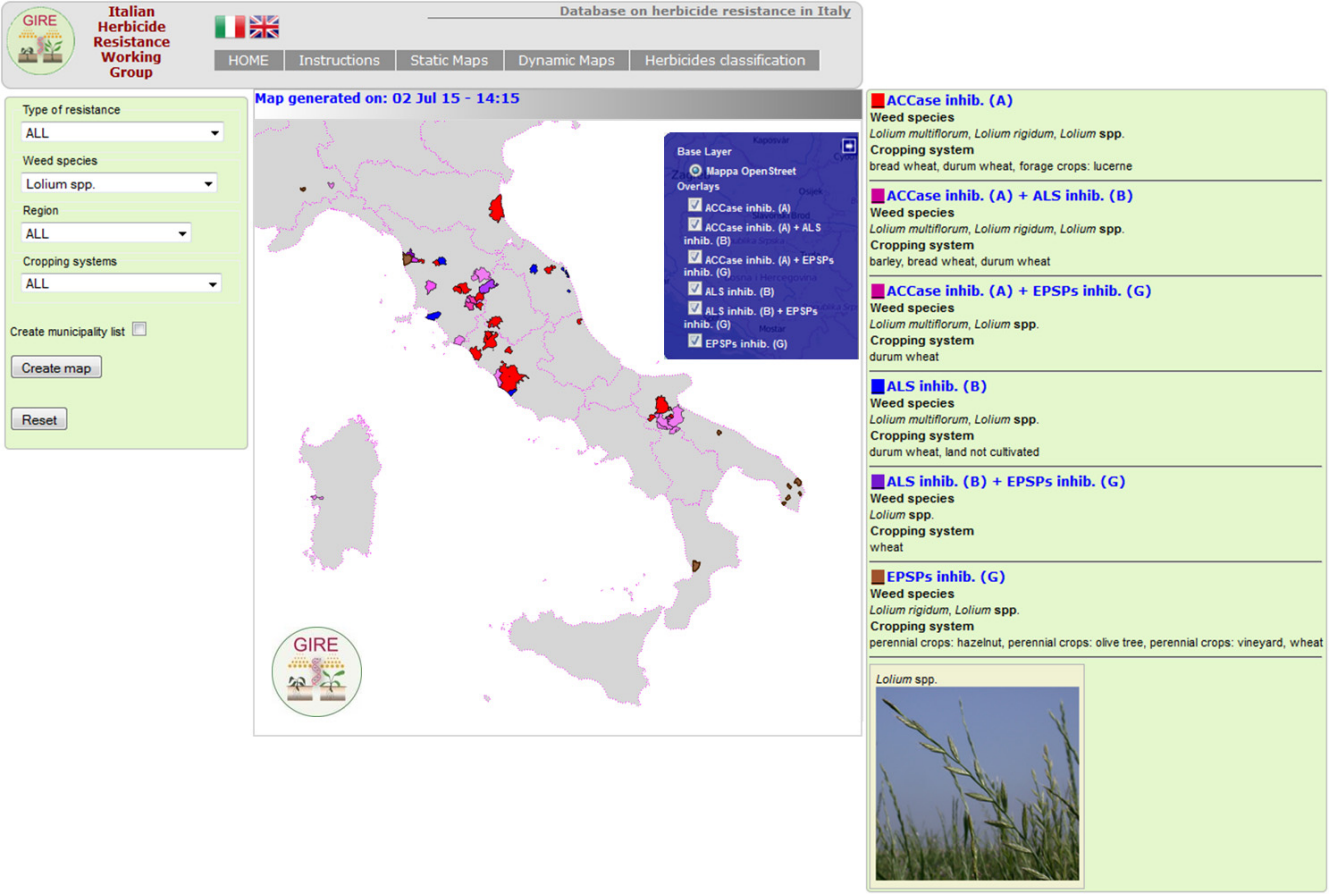
Example of resistant *Lolium* spp. map built using the “dynamic” system. On the right the results of the research where all types of resistance (including multiple ones) are summarized. Each type of resistance is represented by a different color. In the blue box on the map it is possible to tick-untick each type of resistance to simplify and visualize only desired ones. Note: this image is representative of the OpenStreetMap-generated image; for a real representation of this map generated by iMAR go to the link http://5.135.187.175/agri_test/application/views/istruzioni_Fig3.php.

For example, Fig 6 shows all cases of *Lolium* spp. resistant to any herbicide. This map was generated specifying the choice only in the menu “Weed species” and leaving “all” in the other fields. The resistant biotypes of *Lolium* spp. in the different cropping systems, including the multiple resistant cases (not viewable using the static system), are listed to the right of the map. Note that when the option “spp.” is chosen the map includes all the species belonging the selected genus. It is also possible to visualize the multiple resistance cases from the drop-down menu “type of resistance” where all detected biotypes are listed.

## Uses and Perspectives

ICT is playing an increasing role on the rate of adoption of innovation and the dissemination of new knowledge and information on agriculture and environmental issues [42, 43]. This is also true for the crop protection sector [3, 44], which is facing the huge challenge of implementing the new European legislation on the sustainable use of plant protection products [2]. In this context, evolved resistance to pesticides is a key issue. The constant monitoring of the situation and the availability of updated maps of the areas affected by the various resistant biotypes is an important element for devising and implementing efficient resistant management strategies and reducing the impact on the environment.

The large resistance database created by GIRE and constantly updated over the last 15 years is a valuable tool for herbicide resistance management at national level. The iMAR web application makes full use of this database and through open source software allows customized maps to be created of herbicide resistance distribution in Italy, while keeping costs relatively low. This may also allow the system to be extended to European level and, at the same time, adapting the database, the mapping system could be used in other sectors with geographical implications, such as the mapping of invasive plants or pests.

The availability of two systems in the application, i.e. “static” and “dynamic”, makes it suitable for use by a broad range of end-users and for different purposes. For example, simple maps generated by the “static” system can easily be obtained and used by farmers and/or advisors, whereas more complex maps containing several layers with different resistant biotypes can be useful for, and are now being used by, decision makers at various levels, for research [30, 32], and teaching purposes. The Italian Ministry of Agriculture and regional decision makers are already using the maps generated by iMAR and the information available on the GIRE website to make informed decisions on agro-environmental measures and integrated weed management regulations [45]. Given the characteristics described above, iMAR is flexible, easy to use, reliable, always updated and cost-effective. To our knowledge, there are no other similar applications published in the literature and/or publically available online. The English version of iMAR is now also available on the GIRE website.

The proposed system has the potential for further developments that will increase the amount of information available for users. The possibility to add any kind of information to the resistance databases used by iMAR (e.g. mechanism(s) of resistance or cropping practices applied in the years before the appearance of herbicide resistance) or overlay resistance maps with other maps (e.g. soil type, rainfall) in the same web environment could lead to interesting developments. For example, there is little documentation in the literature on the distribution of herbicide resistance genes [25, 46]. Another possible improvement is the inclusion of a search “per year” that would allow the spread of herbicide resistant biotypes over time to be visualized.
